# Amyloid immunotherapy for Alzheimer's disease: the case for cautious adoption

**DOI:** 10.1055/s-0045-1807718

**Published:** 2025-05-09

**Authors:** Jonathan M. Schott, Charles R. Marshall

**Affiliations:** 1 University College London, Queen Square Institute of Neurology, Dementia Research Centre, London, United Kingdom.; 2 Queen Mary University of London, Wolfson Institute of Population Health, Centre for Preventive Neurology, London, United Kingdom.

**Keywords:** Alzheimer Disease, Immunotherapy, Amyloid, Cost-Benefit Analysis

## Abstract

The licensing of lecanemab and donanemab, disease-modifying immunotherapies for Alzheimer's disease (AD) targeting β-amyloid pathology, has been met with difference in opinion about efficacy, adverse effects, and cost-effectiveness. Here we summarize the current situation and make the case for cautious adoption of these treatments into clinical practice. This opinion is predicated on four main observations: 1) these treatments impact the core pathologies of AD and result in meaningful benefits; 2) while adverse effects can be serious, these are proving manageable in clinical practice; 3) upscaling services to deliver these agents is likely to provide wider benefits for diagnosing and treating dementia and facilitating the adoption of future treatments from the dementia drug pipeline; and 4) factoring in both the wider societal cost of care and potential for continued accrual of long term benefits will be likely to bring these treatments within acceptable cost-effectiveness thresholds.

## INTRODUCTION

Alzheimer's disease (AD), the commonest form of dementia, is a devastating, life-limiting illness which has profound implications for individuals, families, and society. Estimates suggest that the world-wide prevalence is now 55 million rising to 139 million by 2050 as the population ages, and those costs exceed $1.3 trillion. 1

At the core of AD is beta-amyloid (β-amyloid) pathology. Numerous lines of evidence suggest that accumulation of toxic β-amyloid species culminating in the deposition of extracellular plaques is an early, upstream, and central pathological process. 2 3

While β-amyloid by itself may cause subtle cognitive problems, 4 a significant proportion of elderly individuals harbor ostensibly asymptomatic β-amyloid pathology for many years. 5 By mechanisms not fully elucidated, but likely to involve inflammation, susceptible individuals then undergo a cascade of pathological processes including the accumulation of tau pathology, synaptic loss, and neuronal cell death leading to the emergence of the progressive cognitive symptoms, increasing dependence, and premature death. 6 7

The centrality of β-amyloid in the pathogenesis of AD has made it a major target for drug development over the last two decades. Initial attempts to induce β-amyloid clearance using a range of different immunotherapies were unsuccessful, variably due to combinations of side-effects, suboptimal therapeutic efficacy, insufficient dosing, and/or inappropriate patient selection, that is, not requiring evidence for β-amyloid pathology at recruitment. 6 However, the situation has changed radically in recent years with the publication of 2 pivotal, positive, phase-3 studies of the monoclonal antibodies lecanemab 8 and donanemab 9 in patients with mild cognitive impairment (MCI) or mild dementia due to AD.

## PROVEN AND POSSIBLE BENEFITS AND POTENTIAL OF AMYLOID IMMUNOTHERAPY

Despite differences in the specific β-amyloid moieties targeted by the two drugs—protofibrils in the case of lecanemab, and plaques for donanemab—the results of the trials are broadly similar. Given intravenously every 2 weeks (lecanemab) or monthly (donanemab) over an 18-month period, both resulted in robust and extensive clearance of β-amyloid; both led to significant slowing of cognitive and functional decline by 35% (donanemab) and 27% (lecanemab) compared with placebo; and both had effects on relevant non-cognitive outcomes including quality of life. 8 9 While the duration of the trials was too short to definitively determine disease-modification effects, these are suggested by changes in other, presumed downstream, biomarkers of AD such as ptau217 (reflecting abnormal phosphorylated tau due to β-amyloid), glial fibrillary acidic protein (GFAP; reflecting astrocytic pathology), and microtubule-binding region of tau containing the residue 243 (MTBR-tau243; reflecting tau tangle pathology). 10 11

## REAL AND POTENTIAL SIDE-EFFECTS OF AMYLOID IMMUNOTHERAPY

Both drugs are associated with significant side-effects. Besides frequent and relatively minor infusion-related reactions, there are potentially more serious side-effects, including amyloid-related imaging abnormalities (ARIAs) comprising brain swelling (ARIA-E), or the emergence of new microhemorrhages (ARIA -H). 12 In the phase-3 clinical trials, ARIA was seen in 21% of patients treated with lecanemab 8 and 39% with donanemab. 8 9 Among patients who developed ARIA, ∼ 80% of cases in clinical trials were asymptomatic and detected on surveillance magnetic resonance imaging (MRI) scans alone; 13 most cases occurred in the first 3 months of dosing; and > 80% resolved spontaneously within 2 to 4 months, 8 9 following which dosing could be safely resumed in the majority. A small but clearly important number of patients did develop symptoms including blurred vision, headaches, unsteadiness, or dysphasias, and rare deaths have now been reported 9 —often but not exclusively in patients with preexisting risk factors. There is now consensus that risk for ARIA is increased in patients with preexisting brain swelling or intracerebral bleeding, and in individuals harboring one, and particularly two, copies of the ApoE-E4 risk allele. 12 These data have informed inclusion/exclusion criteria, requirements for MRI safety monitoring, and genetic testing reflected in appropriate use criteria, 14 and, ultimately, in licensing decisions made by individual countries (see below).

The observation that individuals treated with amyloid immunotherapies as a group have excess brain shrinkage as measured using serial MRI has led to concerns that these drugs might, contrary to predictions, be associated with *excess* neurodegeneration. However, a recent comprehensive review concluded that this excess volume loss is much more likely to be explained by β-amyloid removal. 15 While longer-term follow-up data are required, these authors proposed that this phenomenon be termed amyloid-related pseudoatrophy (ARPA).

## CONTROVERSIES

After more than two decades since the last drug was licensed for AD, one might expect that the emergence of two new therapies with proven efficacy in large well-run phase-3 trials would be greeted with universal enthusiasm. However, the clinical and research community has been far from united, with very robust and heated debate on a range of topics including, but not limited to, clinically meaningfulness, risk-benefit ratio, and cost effectiveness.

There has been much debate about whether the statistically significant observed cognitive benefits based on the absolute changes observed between the treated and placebo, are “ *clinically meaningful* .” Detractors have argued that the observed benefits are, for example, smaller than those seen with standard AD treatments such as acetylcholinesterase inhibitors. 16 Others have argued that these changes *are* clinically meaningful either based on their interpretation of the absolute difference or as demonstrable benefits on non-cognitive measures, including quality of life and career burden. Another argument is that different standards might be appropriate for disease-modifying therapies in which the benefits are hoped to be maintained or to increase over time, noting that some post-hoc analyses of the trial data have suggested the latter, showing divergence of treatment benefits over time. 17 18

At the time of writing, one or both drugs are licensed for use in the United States (US), Japan, China, South Korea, Hong Kong, Israel, Great Britain, and United Arab Emirates, but not in Australia. A reflection of varying and changing opinions is that the European Medicines Agency originally rejected a license for lecanemab, only to reverse this decision on appeal within a few months. 19 Similarly, even in countries endorsing their use, there are differences regarding who is eligible. Thus, in the United Kingdom (UK) and the European Union (EU) neither drug is licensed for use in ApoE4 homozygotes based on a decision that, in this group, the risk (such as ARIA) outweighs the benefit. 19 20 21 22

A related but different question is: When a drug is licensed, who should pay for it? This is a highly complex and very health-system specific calculus based on perceived risk/benefit, system readiness, duration of therapy, and differing views/requirements based on equity and models of payment. Not surprisingly, different healthcare systems have arrived at different conclusions. Thus, in the US, up to 80% of the costs of treatment may be reimbursed by Medicare. 23 Conversely, at the time of writing, in the UK, the National Institute for Health and Care Excellence (NICE) has rejected both drugs for routine use within the free-at-the point of care National Health Service (NHS), citing insufficient cost-effectiveness when the totality of costs—the drug, means of determining inclusion (using positron-emission tomography [PET] scans or cerebrospinal fluid [CSF] analysis), regular infusions over a minimum of 18-months, safety MRI scans, and clinical monitoring—are combined. 24

## WHERE ARE WE NOW?

As a result of the issues outlined above, there remains significant disparity into how these drugs have been used in clinical practice to date. Where these drugs are being prescribed, for example in the US and Japan, experience is slowly growing about their use in a real-life setting. While, thus far, approximately 15 thousand people have been treated worldwide it seems that, at least in the early-adopter and often specialist services offering treatment, it can be done safely and effectively. Experience is informing practicalities of administration and protocol changes to minimize risk. Ongoing use and registries will further inform the longer-term risk/benefits and cost-effectiveness in different populations: it is notable, for instance, that rates of ARIA seem much lower in some countries (Japan) than others (the US). 20

## OUR OPINION

In our view, there can be no doubt that these drugs are effective in slowing cognitive decline, and that they impact on the core pathologies that underpin AD. While side-effects can be significant, both the trial and real-world data suggest that these can be managed safely. It is, of course, important to bear in mind that AD is an inevitably progressive and ultimately fatal illness. There is a risk of excessive medical paternalism in denying patients the opportunity to make an informed decision about risks and benefits. On this basis, we believe strongly that these drugs should be prescribable, and so concur with the positive licensing decisions made by most countries who have thus far opined.

When it comes to who should/will receive these medications, applying licensed inclusion/exclusion and appropriate use criteria to the UK population, it has been estimated that 14% of all those seen in memory services could be eligible. 25 It is unfortunate but perhaps not surprising that despite pleas over many years that preparation to deliver disease-modifying therapies was needed, the health care system in the UK—and in most countries—is simply not ready to deliver these treatments at the scale required. 26 The licensing of lecanemab and donanemab must serve as a wake-up call to urgently upscale our abilities to diagnose and offer novel treatments to patients with dementia; new developments—perhaps most notably the advent of blood tests for AD pathologies—will hopefully smooth this transition. 27 In our view, the reconfiguration of services to deliver these treatments would be likely to result in improvements in diagnosis and management that extend beyond those eligible for β-amyloid-targeting therapies, analogous to the broad and sustained improvements in stroke care that resulted from system reconfiguration to deliver thrombolysis. 28 Moreover, there are around 127 drugs in the development pipeline for AD, and reconfigured services for lecanemab and donanemab will result in much improved system readiness to deliver other emerging treatments in due course. 29

The costs of administering intravenous treatments on a fortnightly or monthly basis and monitoring for side-effects using MRI are clearly a major barrier. The development of next generation immunotherapies that can be administered subcutaneously 30 or via shuttle technologies 31 that require fewer doses and are hopefully safer will hopefully make this more feasible. If longer term data were to show the continued accrual of benefits and resulting reduction in health and social care resource use, then this could also improve the calculated cost effectiveness. However, there are important questions about how cost-effectiveness should be determined when much of the care costs are borne by families and carers and may not, therefore, be adequately factored into health economic models. In the meantime, it is essential that we continue to gain real life experience of how to use the two licensed drugs we have in a range of health care settings and populations. Accordingly, in our view, cautious administration to selected patients in settings able to deliver treatments with appropriate monitoring is the right way forward.
